# Supplementary material for: Comparative analysis of social issues toward medical abortion using mifepristone in South Korea and the United States: Topic modeling and sentiment analysis
Source: PLoS One. 2026 Mar 6;21(3):e0342848. doi: 10.1371/journal.pone.0342848 (PMC12965543; doi:10.1371/journal.pone.0342848)
Supplement: Attachment — Submitted filename: Response to reviewers_final.docx [file pone.0342848.s002.docx]

**ㅠㄱResponse to reviewers**

**Andrea Cioffi**
Academic Editor, Plos One

Dear Cioffi,

We are pleased to resubmit for publication the revised version of PONE-D-25-00622 “Comparative Analysis of Social Issues Towards Medical Abortion Using Mifepristone in South Korea and the US: Topic Modelling and Sentiment Analysis.” We thank you and the reviewers for their critical assessment and thoughtful suggestions of our work. The manuscript has been carefully rechecked, and appropriate changes have been made in accordance with the reviewers’ suggestions. We hope that the revised manuscript is now suitable for publication in *PLOS ONE*.

Sincierely,

Yun-Kyoung Song, Ph.D. (Corresponding author)
College of Pharmacy, The Catholic University of Korea-Sungsim Campus, Bucheon, Gyeonggido, 14662, Republic of Korea

E-mail address: yksong@catholic.ac.kr
Tel: +82-2-2164-5515, +82-10-5099-9741

**Reviewer 1.**

The article is a well-executed and timely study that makes significant contributions to understanding medical abortion discourse in South Korea and the US. However, addressing the outlined limitations and integrating more diverse data and methods could enhance the depth, applicability, and impact of the findings.

1) The reliance on web-scraped news articles from specific platforms like Naver News and Google News may introduce a selection bias, potentially excluding diverse or less mainstream perspectives.

Response:

Thank you very much for your valuable comment.

As you pointed out, our study primarily utilized web-scraped news articles from Naver News and Google News. However, we selected these platforms due to their prominence and representativeness in their respective countries. Specifically, Naver News is the most widely used news aggregator in South Korea, extensively covering diverse news outlets and thus effectively capturing mainstream discourse. Similarly, Google News is the leading news aggregation platform in the United States, encompassing a broad spectrum of major media sources and ensuring the reflection of dominant public narratives.

To clarify and emphasize the representativeness and rationale for choosing these platforms, we have added the following statement in the Methods section of the revised manuscript:

*To ensure the reliability and relevance of the collected articles, only articles from registered news media sources indexed by Google News and Naver News were included. Naver News was considered for South Korea owing to its dominant position in aggregating nearly all major domestic news outlets [22], and Google News was selected for the US because of its extensive and representative aggregation of leading American news sources [23]. Both platforms have news policies [24–25] to ensure that the content they provide comes from media outlets that adhere to best practices.*

2) The dataset includes articles from 2006 to 2022, which may overlook temporal shifts in discourse, especially after pivotal legal or policy changes.

Response:

We are grateful for the valuable feedback. The study period (2006–2022) was strategically selected to capture critical legal and policy shifts related to medical abortion. We have clarified the rationale for this choice in the discussion section of the revised manuscript as the following:

*Additionally, our analysis spans news articles between 2006 and 2022, potentially introducing a time-trend bias, as public discourse on media perspectives possibly evolved over this period. However, the study period (2006–2022) was specifically selected to capture critical legal and policy shifts related to abortion, enabling a thorough analysis of how media discourse adapted to these temporal shifts. This period saw global and regional regulatory milestones regarding mifepristone. In February 2006, the Federal Parliament of Australia overturned the Harradine Amendment—a legislative restriction that had previously limited the availability of mifepristone [41]. Moreover, in South Korea, the selected timeframe captured the 2017 Blue House petition demanding reconsideration of abortion regulations, the Constitutional Court’s landmark 2019 ruling deeming the existing abortion ban unconstitutional, and the subsequent abolishment of abortion restrictions in January 2021. The observed increase in discourse following Hyundai Pharmaceutical’s initial submission for mifepristone approval in 2021 further validates that the analyzed period encompassed key events that likely influenced media narratives. Thus, the extended analysis period adequately addressed potential concerns about overlooking temporal shifts in media discourse, illustrating how public narratives evolved alongside these landmark events. Future research could address these limitations by incorporating more news sources, employing dynamic topic modeling to capture temporal trends, and integrating qualitative methods.*

3) While the study attempts to adapt sentiment analysis tools for Korean and English texts, the effectiveness of these tools in capturing cultural nuances and contextual meanings remains unclear. Misinterpretation of sentiments could affect the validity of the results.

Response:

We sincerely appreciate the reviewer’s insightful comment regarding potential limitations associated with the sentiment analysis tools used for Korean and English texts. As pointed out, there is a possibility of misinterpretation due to cultural nuances and contextual differences across languages. We fully acknowledge this limitation, and we have explicitly addressed this point in the Discussion section of our revised manuscript. Specifically, we added references that demonstrate the validity and common usage of these sentiment analysis tools in cross-cultural text mining studies, while simultaneously emphasizing the importance of cautious interpretation when applying lexicon-based approaches to culturally distinct datasets.

*We acknowledge a potential limitation regarding the effectiveness of lexicon-based sentiment analysis tools in accurately capturing the cultural nuances and contextual meanings inherent in Korean and English texts. While these tools have been validated and commonly used across diverse linguistic and cultural contexts in previous research [38–40], there remains a possibility of misinterpretation when applied to culturally distinct datasets. To mitigate it, the researchers involved in this study who are proficient in both languages cross-validated and any disagreements regarding contextual interpretation beyond automated sentiment analysis outputs were resolved through discussion. Nevertheless, our sentiment analysis results should be interpreted with caution, and future research should consider additional qualitative analyses or culturally customized lexicons to better account for language-specific subtleties.*

4) The exclusive focus on news articles neglects other influential media forms, such as social media or public opinion surveys, which might provide a broader understanding of societal attitudes.

Response:

Thank you very much for highlighting this important point regarding our exclusive reliance on news articles. We fully acknowledge that incorporating social media or public opinion survey data could offer additional valuable perspectives on societal attitudes toward medical abortion. However, our research specifically aimed to capture institutional media representations of regulatory, societal, and policy discussions rather than individual, potentially biased viewpoints that frequently appear in social media. We have clarified this focus in the revised discussion section, emphasizing that social media data, while valuable, could introduce individual-level biases and distortions. Nevertheless, we suggest that future studies incorporate social media or public opinion data as complementary resources to expand and enrich the understanding of societal attitudes towards medical abortion.

*This study had some limitations. While various big data open-sources are available for text mining, our analysis relied on web-crawled news articles from specific platforms and periods, which may not capture the full spectrum of media discourse on mifepristone. This study analyzed news articles to accurately capture formal media representations of regulatory, societal, and policy issues regarding medical abortion. Although leveraging social media content for the analysis could provide additional insights, individual opinions expressed through social media platforms usually introduce significant biases and distortions, precluding the accurate assessment of institutional discourse.*

**Reviewer #2:**

I read with great interest your article on abortion. This study will use AI-driven text analysis (topic modeling and sentiment analysis) to examine and compare how abortion with mifepristone is discussed in South Korea and the US. It will analyze social issues, legal debates, cultural attitudes, and public sentiment surrounding medical abortion, offering insights into how the discourse varies across different legal, ethical, and cultural landscapes. My concern is that if there is no human input to choose which articles to be included in the analysis, then it may mean that you are including everything which may not reflect the actual social, cultural and legal attitudes. google and other sites are full of non-sense information. I am not sure if we can draw any valuable conclusions if we are not fully aware of what we are analysing.

Response:

We appreciate the reviewer’s important comment. The description regarding sorting out irrelevant or duplicate articles was inadvertently omitted in the initial manuscript. We have now clearly described that only articles from registered and reputable news media indexed by Google and Naver were utilized, and irrelevant or duplicated articles were systematically excluded through manual review and automated deduplication processes. This clarification has been added to the Methods section in the revised manuscript. We sincerely appreciate your insightful suggestion.

*To ensure the reliability and relevance of the collected articles, only articles from registered news media sources indexed by Google News and Naver News were included. Naver News was considered for South Korea owing to its dominant position in aggregating nearly all major domestic news outlets [22], and Google News was selected for the US because of its extensive and representative aggregation of leading American news sources [23]. Both platforms have news policies [24–25] to ensure that the content they provide comes from media outlets that adhere to best practices.* *Duplicate articles and those irrelevant to the study’s context were identified and excluded through manual sorting and automated deduplication processes.*

**Reviewer #3:** 
I sincerely believe this is an excellent piece of work, carefully designed, well-executed, and meaningful in its scope and contribution.

Abstract
The abstract serves as the gateway and first impression of the manuscript. I recommend that you make it more comprehensive and inclusive, offering a clear overview of the study's objectives, methods, key findings, and conclusions. This will help readers grasp the essence of your work at a glance.

Response:

Thank you very much for your valuable feedback on our abstract.

Following your suggestion, we have revised the abstract to clearly and comprehensively present the study's objective, methods, key findings, and conclusions. We believe this updated version effectively summarizes the essence of our research and provides readers with a clearer overview.

Background
Well done. However, to strengthen this section, I suggest expanding on the research problem and the significance of the study. Additionally, it would be valuable to discuss the global use and prevalence of mifepristone. Including relevant data from the World Health Organization (WHO) could provide useful context and reinforce the importance of your research.

Response:

Thank you for your valuable comments on strengthening our introduction.

As suggested, we have expanded our introduction to clearly articulate the research problem and significance of this study. Additionally, we included relevant data from the World Health Organization (WHO) to provide global context regarding the prevalence and recommended use of mifepristone, thereby reinforcing the importance and relevance of our research.

The revised introduction section is presented below.

*Mifepristone, a common oral abortifacient, is legally available in numerous countries, and the World Health Organization (WHO) has endorsed the combined use of mifepristone and misoprostol as safe, effective, and highly acceptable regimen for early abortion—defined as abortion during the first trimester [3]. In 2019, the WHO moved these medications from the “complementary” to the “core” list of its Essential Medicines List, given their critical role in global reproductive health [4].*

*Following these recommendations, the global prevalence of medical abortion has increased, particularly in high-income countries [5]. By 2017, medical abortion comprised more than one-half of all abortion provisions in at least 24 high-income nations, with high proportions in Sweden, England, and the United States (US). In the US in 2023, approximately 63% of all abortions were medication-based abortions [5]. Nevertheless, regulatory approval and access to mifepristone vary among countries; for instance, despite its use in the US [5–7], it remains unapproved in South Korean, resulting in limited availability of medical abortion [8].*

Methods
• This section would benefit from a more detailed explanation of the platform selection process for data extraction. Please clarify the steps and procedures followed, including the criteria for inclusion and exclusion of data sources.

Response:

Thank you for this insightful suggestion.

We have now clarified and expanded upon the platform selection process in the Methods section, specifying detailed criteria used for including or excluding data sources. This revision highlights the rationale behind the selection of Naver News and Google News platforms, including their representativeness and comprehensiveness, and explicitly describes the inclusion/exclusion criteria employed during article collection.

The revised Methods section is provided below.

*To ensure the reliability and relevance of the collected articles, only articles from registered news media sources indexed by Google News and Naver News were included. Naver News was considered for South Korea owing to its dominant position in aggregating nearly all major domestic news outlets [22], and Google News was selected for the US because of its extensive and representative aggregation of leading American news sources [23]. Both platforms have news policies [24–25] to ensure that the content they provide comes from media outlets that adhere to best practices.* *Duplicate articles and those irrelevant to the study’s context were identified and excluded through manual sorting and automated deduplication processes.*

Methods
• Furthermore, a brief but clear description of your measurement procedures would enhance the methodological transparency of the study.

Response:

Thank you for your suggestion. We fully agree that including a brief yet clear explanation of the measurement procedures would enhance the methodological transparency of our study. The revised Methods sections reflecting your comment is provided below.

*The study comprised three stages: (1) data collection and preprocessing, (2) topic modeling using Latent Dirichlet Allocation (LDA), and (3) sentiment analysis using lexicon-based methods. The outline of the text analysis pipeline is described in Fig 1.*

Conclusion
Since this is the final impression of your work, I suggest refining it slightly. Highlight the key findings, emphasize their wider relevance, and reinforce the significance of the study. A strong conclusion provides a sense of closure and impact for the reader.

Response:

We have refined the conclusion to clearly highlight the key findings, emphasize their broader implications, and reinforce the importance of our research. The revised conclusion is presented below.

*This study identifies differences in media discourse on medical abortion using mifepristone between South Korea and the US, reflecting differences in the number of articles, key topics, and distinctive patterns of sentiments in each country. The topic modelling results showed that the Korean news focuses on regulatory challenges, safety concerns, and social perceptions, whereas the US coverage underscores clinical aspects and access-related debates. Our findings reveal differing public concerns and interests between the two countries, which may assist policymakers and healthcare providers in developing strategies to navigate complex societal dynamics surrounding medical abortion.*

References
Some references appear not to fully align with the journal’s formatting guidelines. Please ensure that all citations strictly follow the required style.

Response:

Thank you for highlighting the formatting issue. We carefully reviewed and revised references to strictly adhere to the PLOS One guidelines (Vancouver style). All citations have been reformatted and verified to ensure complete compliance with journal standards.

**Reviewer #4:**

I would like to thank the authors for the opportunity to review this manuscript. The study presents an interesting comparative analysis of media discourse surrounding mifepristone and medical abortion in South Korea and the United States using text mining techniques. However, there are several areas that would benefit from revision

1) In the introduction: what are the specific gaps in knowledge that this study aims to address. And why these two countries?

Response:

Thank you for this valuable suggestion. We fully agree that explicitly clarifying the research gaps and the rationale behind selecting these two countries strengthens our manuscript. Accordingly, we have clearly articulated the specific knowledge gaps our study addresses and explicitly justified the choice of these two countries in the revised Introduction as follows.

*Yet, despite these methodological advances, there remains a gap in comparing how media discourse on medical abortion differs between countries with contrasting regulatory and cultural contexts, such as South Korea and the US. South Korea represents a restrictive environment characterized by limited access, significant cultural stigma, and ongoing regulatory debates, whereas the US context is marked by broader regulatory approval, active public discourse, and evolving policy initiatives aimed at expanding access. Addressing this gap is crucial, as understanding how divergent societal and regulatory contexts influence media discourse may inform culturally sensitive and evidence-based reproductive health policies.*

2) The data collection process lacks transparency regarding the search terms used for web crawling. The authors mention using Google News and Naver News but do not specify the exact search queries employed.

Response:

Thank you for your valuable comments. As suggested, we have clarified the specific research terms used for collecting news articles from Google News and Naver News in the methods section of the revised manuscript.

*News articles related to mifepristone, published between January 1, 2006, and December 31, 2022, were collected from Google News [20] and Naver News [21] using web scraping techniques, the Python library: Beautifulsoup. The search words included “Mifepristone,” “Misoprostol,” “Mifegyne,” and “RU-486” in Korean news platforms and in English for the US news platforms.*

3) The authors should provide more information about the criteria used for including or excluding news articles. Was there any screening process to ensure the articles were specifically focused on mifepristone rather than abortion more broadly?

Response:

Thank you for highlighting the important point regarding our data inclusion and exclusion criteria. To ensure that our collected data specifically focused on mifepristone rather than abortion more broadly, we searched only for articles and titles explicitly containing predefined keywords ("Mifepristone," "Misoprostol," "Mifegyne," "RU-486" in English; "미페프리스톤," "미프진," "미프지미소" in Korean). By clearly restricting our researches to these keywords, we aimed to systematically exclude irrelevant or overly general articles from our analysis. We have clarified this methodology explicitly in our revised Methods section under the sub-section called “Data collection and preprocessing”.

*News articles related to mifepristone, published between January 1, 2006, and December 31, 2022, were collected from Google News [20] and Naver News [21] using web scraping techniques, the Python library: Beautifulsoup. The search words included “Mifepristone,” “Misoprostol,” “Mifegyne,” and “RU-486” in Korean news platforms and in English for the US news platforms. On Google News, further targeted searches were conducted to retrieve relevant articles from various news media websites, such as the New York Times, and the Washington Post. To ensure the reliability and relevance of the collected articles, only articles from registered news media sources indexed by Google News and Naver News were included. Naver News was considered for South Korea owing to its dominant position in aggregating nearly all major domestic news outlets [22], and Google News was selected for the US because of its extensive and representative aggregation of leading American news sources [23]. Both platforms have news policies [24–25] to ensure that the content they provide comes from media outlets that adhere to best practices. Duplicate articles and those irrelevant to the study’s context were identified and excluded through manual sorting and automated deduplication processes.*

4) The significant disparity in sample size between Korean (7,938) and US (650) articles raises concerns about comparability. The authors should address how this imbalance might affect the interpretation of results and whether any normalization techniques were applied to account for this difference.

Response:

Thank you very much for highlighting the significant numerical imbalance between the South Korean (7,938) and US (650) news articles. We acknowledge this disparity and recognize the potential concern regarding the comparability of our analysis. However, given our qualitative analytical approach focused on thematic exploration rather than direct numerical comparisons, normalization or other quantitative balancing methods were not applied. We have explicitly addressed and clarified this point as a limitation in the revised manuscript’s Discussion section, explaining why this imbalance does not significantly impact the interpretative validity of our thematic and sentiment analyses.

*Notably, there was a disparity in the volume of news articles, as the South Korean dataset contained 7,938 articles vs. 650 articles for the US. This contrast can be attributable to the media attention in South Korea, largely driven by domestic regulatory events, such as the abolition of abortion restrictions in 2021 and the subsequent regulatory debates concerning mifepristone approval from Ministry of Food and Drug Safety. Although we cannot weigh the relative prominence of thematic content based on the article numbers, it is worth emphasizing that our primary goal was to provide qualitative overview and comparison of predominant themes and sentiments rather than conduct direct quantitative comparisons in publications between the two countries. Accordingly, we did not normalize the number of articles for the analysis. This decision stems from our view that the numerical contrast inherently represents distinct patterns of public interest and discourse across countries, and normalization could potentially introduce bias. TF-IDF scores were computed separately for each country’s corpus. Because TF-IDF incorporates term frequency normalized within individual documents and an inverse document frequency that accounts for overall term dispersion, differences in corpus size do not bias the resulting term-weighting. Consequently, we are confident that this numerical imbalance will not substantially affect the interpretative validity of our thematic and sentiment analyses.*

5) Figure 1 (Pipeline of the text analysis) is referenced but not adequately described in the text.

Response:

We appreciate your helpful suggestion. To address your concern and enhance clarity, we have now added description of Figure 1 in the revised Methods section.

*The study comprised three stages: (1) data collection and preprocessing, (2) topic modeling using Latent Dirichlet Allocation (LDA), and (3) sentiment analysis using lexicon-based methods. The outline of the text analysis pipeline is described in Fig 1.*

6) grammatical errors and awkward phrasings throughout the manuscript; e.g:
- line 41: "including and is extensively prescribed" → delete "including."
- Line 68: "Comparative analyses of news content between countries can provide" → "Comparative analyses of news content can provide."
- Page 1, Abstract: "distinct pattern" should be "distinct patterns".

Response:

We sincerely apologize for our oversight. We have carefully reviewed the manuscript in detail and have corrected errors and improved phrasing throughout. Thank you.

7) The abbreviations should be defined at first use and then used consistently throughout the manuscript.

Response:

Thank you very much for highlighting this issue regarding abbreviations. We apologize for our oversight in not clearly defining abbreviations upon their first use and ensuring consistency throughout the manuscript. We have thoroughly reviewed and revised the manuscript to ensure that all abbreviations are explicitly defined at first occurrence and consistently used thereafter.
