# Supplementary material for: Comparative analysis of social issues toward medical abortion using mifepristone in South Korea and the United States: Topic modeling and sentiment analysis
Source: PLoS One. 2026 Mar 6;21(3):e0342848. doi: 10.1371/journal.pone.0342848 (PMC12965543; doi:10.1371/journal.pone.0342848)
Supplement: Attachment — Submitted filename: Response to reviewers_rev2-final.docx [file pone.0342848.s003.docx]

**Response to reviewers**

**Andrea Cioffi**
Academic Editor, Plos One

Dear Cioffi,

We are pleased to resubmit for publication the revised version of PONE-D-25-00622R1 “Comparative Analysis of Social Issues Towards Medical Abortion Using Mifepristone in South Korea and the US: Topic Modelling and Sentiment Analysis.” We thank you and the reviewers for their critical assessment and thoughtful suggestions of our work. The manuscript has been carefully rechecked, and appropriate changes have been made in accordance with the reviewers’ suggestions. We hope that the revised manuscript is now suitable for publication in *PLOS ONE*.

Sincierely,

Yun-Kyoung Song, Ph.D. (Corresponding author)
College of Pharmacy, The Catholic University of Korea-Sungsim Campus, Bucheon, Gyeonggido, 14662, Republic of Korea

E-mail address: yksong@catholic.ac.kr
Tel: +82-2-2164-5515, +82-10-5099-9741

**Reviewer 1.**

The revised manuscript effectively addresses the prior concerns and now presents a well-structured, ethically sensitive, and scientifically sound analysis with just minor refinements as noted below:

1) Ethical Clarity: You mention ethics approval and waiver of consent, but do not explicitly state how sensitive abortion data were handled (e.g., anonymisation, database separation, encryption).

Response:

Thank you very much for your comments with ethical clarity.

We agree with the importance of clarifying the potential sensitivity regarding the data. However, our study did not involve any human subjects or personally identifiable information. The dataset we used consisted exclusively of publicly available news articles crawled from online news platforms. No individual-level or sensitive health data were included, and all analyses were performed on anonymized textual data in aggregate form.

We have added a clarifying statement in the Methods section as the following:

*This study was a retrospective comparative analysis of published news articles using a text-mining approach. As the study was conducted using publicly available online news articles without any non-identifiable data and that did not involve human participants, no sensitive or confidential data were included in the dataset, and all analyses were conducted on aggregated, anonymized text corpora. Therefore, it was exempted from ethical review by the Institutional Review Board (IRB) of Daegu Catholic University (IRB No. CUIRB-2022-E004).*

2) Cultural Context (Discussion section): A brief acknowledgement that the findings may not be generalizable to settings where abortion is legally restricted or stigmatised would enhance the discussion’s depth.

Response:

We appreciate the suggestion. We agree that the generalizability of our findings may be limited based on legal restrictions or social stigmas. While we already have noted the need for cultural sensitivity in interpreting sentiment analysis results, we further expanded this part in Discussion section to explicitly acknowledge that media discourse and public sentiment toward medical abortion can be shaped by distinct cultural, legal, and ethical environments as suggested.

*It should also be noted that the generalizability of these findings may be limited as media narratives and public sentiment towards medical abortion are influenced by the unique cultural, legal, and ethical environment.*

3) Clinical Significance: The adjusted odds ratios for biochemical pregnancy and clinical pregnancy are slightly reduced but not statistically significant. Consider adding a sentence to discuss whether this might still be clinically meaningful or merits further investigation in a larger cohort.

Response:

We appreciate the reviewer’s thorough evaluation. We revised the discussion section as follows:

*Sentiment analysis on news coverage surrounding mifepristone in each country provided insights into the public perceptions. The US articles displayed a polarized pattern, with strongly positive sentiments associated with the term 'treatment' and negative sentiments linked to 'restrictions'. Other terms, such as 'women,' remained relatively neutral in the US dataset. In contrast, Korean articles demonstrated more varied sentiment distributions. Terms such as 'legalize', 'petition', and 'abolish' conveyed negative sentiments, reflecting both the restrictive environment and moral concerns surrounding abortion, while words like 'overseas,' 'treatment,' and 'safety' elicited positive sentiments. Notably, the term 'abortion' was associated with near-neutral sentiment in Korean texts, potentially indicating* ***changing public attitudes and reflecting ongoing challenges in the societal acceptance of medical abortion in South Korea [13,32].*** ***The neutrality associated with the word 'permit' and slight negativity with terms such as 'legalize' and 'sale' in Korean articles highlight the underlying ambivalence and moral unease within public discourse. Such sentiment patterns suggest persistent societal resistance and caution toward the acceptance of medical abortion, reflecting broader apprehensions about the moral and health implications of expanding access to abortifacient drugs. These findings underscore the need for prudent regulatory approaches that prevent the normalization of medically and morally contentious practices. This contrasts with countries such as the US and Canada, where extensive evidence has contributed to widespread regulatory acceptance and clinical recognition of the effectiveness of mifepristone [36,37]. Furthermore, given the marginally neutral but somewhat negative connotation of the term ‘labor-inducing agent’, further large-scale cohort studies are warranted in countries where mifepristone is used, to assess its long-term safety in women. These findings emphasize the need for careful, value-sensitive and evidence-based policies that respect prevailing ethical perspectives while addressing emerging needs for safe clinical practices [38].***

**Reviewer #5:**

I would like to sincerely thank the Editor for the opportunity to review a manuscript submitted to such a highly esteemed journal. It is an honour for me. It has been a pleasure to read this article, which I found to be well-structured and highly original in both its methodological approach and thematic focus. Nevertheless, I believe that some revisions are necessary before the manuscript can be recommended for publication. I hope that the following suggestions will support the authors in strengthening their work and guiding their revisions.

The Introduction is solid, well-documented, and clearly structured, with a strong rationale for the study. However, improvements in language style, clarity, and conciseness would enhance the manuscript’s readability and impact, particularly for an international audience. Additionally, several grammatical issues require attention before publication. For instance, the phrase “in South Korean” should be corrected to “in South Korea,” and expressions such as “concerns persist safety of mifepristone” are grammatically problematic. Terms such as “medial abortion” also need revision for accuracy. Moreover, the legal and regulatory references appear somewhat imbalanced: while the US context is discussed in detail, the Korean legal framework receives more limited attention. A more thorough and globally contextualized background would be beneficial. In particular, integrating European examples would offer a more comprehensive overview of international approaches to medical abortion—especially since the conclusions highlight global policy implications. To that end, I recommend including references such as: https://pubmed.ncbi.nlm.nih.gov/34897104/ https://pubmed.ncbi.nlm.nih.gov/12831608/ https://pubmed.ncbi.nlm.nih.gov/12137129/ These sources can enrich the comparative dimension of the analysis and support a more robust global framing.

Response:

We appreciate deeply your valuable comments and constructive suggestions. We have thoroughly revised the introduction for improved clarity, language, and conciseness, correcting all grammatical issues. Additionally, we have revised the Introduction overall to ensure a more balanced discussion of both countries. To strengthen the global perspective, relevant European examples were incorporated with proper references cited in both Introduction section as follows.

***Abortion remains a socially divisive issue, balancing women’s reproductive rights against moral and ethical opposition [1]. Access to abortion services varies globally depending on legislation, social values, and healthcare policies. Medical abortion using mifepristone has raised ongoing debates regarding its moral legitimacy and potential health risks, although often described as a less invasive alternative to surgery [2]. Approved by the United States (US) Food and Drug Administration (FDA) in 2000 after several decades of use in European countries such as France, the United Kingdom. Italy and Sweden, mifepristone is now available in numerous countries, following the World Health Organization’s (WHO) endorsement of its use in combination with misoprostol as an early abortion regimen [3–6]. Prevalence of medical abortion has increased worldwide, comprising more than half of all abortions in high-income countries. In the US, despite the FDA’s regulatory oversight and the adoption of Risk Evaluation and Mitigation Strategy, reports of adverse events such as severe bleeding, ectopic pregnancy, and systemic infections persist [7–11]. In contrast, South Korea has yet to approve mifepristone despite the 2019 Constitutional Court ruling decriminalizing abortion up to 14 weeks of gestation [12,13]. Cultural stigma, conservative values, and limited public discourse on reproductive ethics continue to restrict access, underscoring the need for cautious, evidence-based policymaking than balances women’s health with broader moral and societal consideration [13].***

***Media discourse plays a pivotal role in shaping public attitudes and policy debates on contested topics such as abortion [14,15]. Coverage of medical abortion and mifepristone reflects prevailing societal attitudes, cultural beliefs, and political contexts. Comparative analyses of news content across countries can illuminate how differing moral climates and policy priorities generate divergent narratives [16,17]. Such analysis can elucidate underlying factors that contribute to disparate approaches towards medical abortion, thereby supporting the evidence-based communication and policy strategies. In the digital era, computational text-mining techniques such as topic modelling and sentiment analysis enable systemic exploration of dominant themes, sentiment trends, and linguistic patterns in media representations of health issues [18]. However, limited research has compared media discourse on medical abortion across countries with contrasting regulatory and cultural contexts, notably South Korea and the US. Understanding these differences is crucial to evaluating how societal values and policy frameworks shape media narratives, thereby informing culturally sensitive and empirically grounded reproductive health policymaking.***

*Therefore, this study aims to comprehensively compare news media coverage in South Korea and the US through topic modeling and sentiment analysis, identifying predominant themes and sentiment patterns* ***surrounding medical abortion with mifepristone. By clarifying how distinct national contexts influence media narratives and public perceptions, this research seeks to provide critical insights to guide policymakers, healthcare providers, and advocacy groups in developing culturally informed reproductive health policies.***

The Methods section is clearly structured and methodologically sound. However, unless I have overlooked it, the initial number of articles retrieved per country (prior to filtering or selection) is not explicitly stated—this detail would improve transparency. Additionally, I would suggest adding a brief note regarding the reproducibility of the research process (e.g., availability of code, corpora, or full protocol), which is particularly important in computational text analysis.

Response:

We sincerely appreciate your valuable comments regarding methodological transparency and reproducibility. As noted in the revised manuscript, we included only news articles that contained *mifepristone*-related keywords in either the title or the main text. Duplicate articles were automatically removed using a pre-specified script, after which all remaining articles were manually reviewed; no additional exclusions were necessary. Therefore, the 7,938 Korean and 650 U.S. news articles reported in the manuscript represent the final datasets used for the analysis.

Regarding reproducibility, we acknowledge that the exact number of articles or corpora may vary in the future if certain news articles are deleted by publishers or if platform crawling policies are modified. Nevertheless, to enhance research transparency, we have added a statement at the end of the manuscript indicating that the analysis code is available from the authors upon reasonable request.

For greater clarity, the manuscript has also been revised as follows:

*The search included news articles that contained the keywords “mifepristone,” “misoprostol,” “Mifegyne,” or “RU-486” in either the title or the main text. On Korean platforms, these keywords were searched in Korean, while English terms were used for the U.S. platforms……. There were no irrelevant articles identified after manual sorting.*

***Data Availability Statement***

*The datasets analyzed in this study were derived from publicly available online news articles retrieved from Google News and Naver News between January 2006 and December 2022. Both the processed datasets and the analysis code used in this study are available from the corresponding author upon reasonable request. However, the exact number of accessible articles or the composition of the corpora may vary in the future depending on changes in news outlet availability or platform crawling policies.*

The Results are presented in a clear and orderly manner, and the comparative analysis between South Korea and the US is effective. To further support interpretation, I suggest including a short summary section that explicitly highlights the key differences observed between the two countries in terms of themes and sentiments.

Response:

Thank you for this helpful suggestion. We agree that adding a concise summary of the key differences would enhance readers’ understanding of the results. Since the Results section is organized by four analytical components with specific subheadings, adding an additional paragraph at the end could disrupt the structural flow and cause redundancy. Therefore, we have incorporated a clear summary paragraph at the beginning of the Discussion section as follows.

*This study examines the social context of mifepristone in South Korea and the US through an analysis of news media discourse using medical informatics methodology. Leveraging analytical methods including text mining, topic modeling, and sentiment analysis, we identified prominent topics, underlying sentiments, and word associations that illustrate public attitudes and policy discourse regarding medical abortion within regulatory and healthcare contexts.* ***The findings revealed media coverage in South Korea primarily emphasized the regulatory approval process, safety concerns, and social controversies. In contrast, the US media discourse was characterized by legal debates, clinical applications, and accessibility, with more polarized sentiment patterns.***

In the Discussion, two aspects warrant careful attention: Avoid implying causal relationships that are not empirically grounded. For example, the assumption that illegal online trade results directly from regulatory restrictions should be carefully qualified as a possible causal link or interpretative hypothesis, unless further evidence is provided. The influence of media narratives on public opinion is mentioned but not sufficiently explored. A brief reflection on how and why media framing can shape public understanding—supported by relevant literature—would add theoretical depth and policy relevance to the discussion.

Response:

We sincerely appreciate this insightful comment. In response, we have carefully reviewed and revised the Discussion section, integrating relevant literatures as follows.

*Furthermore, our study noted the illegal online trade of mifepristone frequently reported in Korean news coverage.* ***Such reporting may suggest possible unintended associations between stringent regulatory controls and unsafe practices and misuse, raising public health and safety concerns [34]. These observations underscore the importance of developing balanced, evidence-based regulatory frameworks prioritizing public health and moral responsibility. Despite the role of legal changes in abortion debates, the word ‘law’ did not appear among the terms with high TF-IDF values in Korean articles. This omission may indicate a strong media focus on practical considerations related to the introduction of medical abortion rather than on legal debates surrounding the abolition of abortion laws. The media narratives are becoming increasingly important in shaping public belief and opinions, ultimately contributing to social change [14,35]. This practice may indicate the evolving nature of public discourse on abortion in South Korea, warranting further investigation into how media framing shapes the public understanding of complex health concerns.***

The Conclusion successfully summarizes the findings but remains rather general. It would benefit from being more precise and action-oriented, particularly by offering concrete recommendations. For example, the authors could suggest how media coverage might adopt a more balanced and informative narrative regarding medical abortion, or how policymakers might engage with media discourses more strategically.

Response:

We appreciate the reviewer’s constructive feedback regarding the conclusion. We revised the Conclusion section as follows.

*This study identifies differences in media discourse on medical abortion using mifepristone between South Korea and the US, reflecting differences in the number of articles, key topics, and distinctive patterns of sentiments in each country.* ***The Korean news coverage focused on regulatory challenges, safety concerns, and social perceptions, whereas the US coverage underscored clinical aspects and access-related debates.*** *Our findings reveal differing public concerns and interests between the two countries, which may assist policymakers and healthcare providers in developing strategies to navigate complex societal dynamics surrounding medical abortion* ***through media discourses. Furthermore, it is essential for the media to adopt more balanced and informative narratives when addressing culturally and ethically sensitive topics, including medical abortion.***
