# Supplementary material for: Comparative analysis of social issues toward medical abortion using mifepristone in South Korea and the United States: Topic modeling and sentiment analysis
Source: PLoS One. 2026 Mar 6;21(3):e0342848. doi: 10.1371/journal.pone.0342848 (PMC12965543; doi:10.1371/journal.pone.0342848)
Supplement: Attachment — Submitted filename: Response to reviewers_rev3.docx [file pone.0342848.s004.docx]

**Response to reviewers**

**Andrea Cioffi**
Academic Editor, Plos One

Dear Cioffi,

We resubmit for publication the revised version of PONE-D-25-00622R2 “Comparative Analysis of Social Issues Towards Medical Abortion Using Mifepristone in South Korea and the US: Topic Modelling and Sentiment Analysis.” We thank you and the reviewers for their critical assessment and thoughtful suggestions of our work. We sincerely apologize to Reviewer #5 for the inconvenience caused by the lack of clarity in our previous revision. We fully understand that the difficulty in identifying the specific changes may have created unnecessary burden during the review process.

We hope that the revised manuscript is now suitable for publication in *PLOS ONE*.

Sincierely,

Yun-Kyoung Song, Ph.D. (Corresponding author)
College of Pharmacy, The Catholic University of Korea-Sungsim Campus, Bucheon, Gyeonggido, 14662, Republic of Korea

E-mail address: yksong@catholic.ac.kr
Tel: +82-2-2164-5515, +82-10-5099-9741

**Reviewer #5:**

I am unable to clearly identify the revisions made in response to my previous comments. Although the authors provided a point-by-point reply, these responses do not always correspond in a clear and consistent manner to changes in the main text.

I therefore kindly ask the authors to submit a newly revised version of the manuscript, in clean format, in which only the additions, modifications, and new references introduced in response to the previous review are clearly highlighted-for example, marked in red-so as to allow a more accurate assessment of the adequacy of the revisions undertaken.

Response:

We sincerely thank Reviewer #5 for the careful reading of our manuscript and for pointing out the difficulty in identifying the exact revisions made in the previous round. We deeply appreciate the reviewer’s patience and the opportunity to further clarify and improve the transparency of our revision process.

In this resubmission, we have taken several steps to ensure that all revisions are fully traceable, clearly highlighted, and directly matched to each comment. In addition, as we substantially revised the Introduction to improve clarity and coherence, only newly added text has been highlighted in red.

| **Reviewer’s Comment** | The Introduction is solid, well-documented, and clearly structured, with a strong rationale for the study. However, improvements in language style, clarity, and conciseness would enhance the manuscript’s readability and impact, particularly for an international audience. Additionally, several grammatical issues require attention before publication. For instance, the phrase “in South Korean” should be corrected to “in South Korea,” and expressions such as “concerns persist safety of mifepristone” are grammatically problematic. Terms such as “medial abortion” also need revision for accuracy. | |
| --- | --- | --- |
| **Before (Revision 1)** | | **After (Further revised version)**  We have extensively revised the overall narrative and structure of the introduction to improve clarity, accuracy, and coherence in response to the reviewer’s comments. |
| **[Location: Introduction section]**   - for instance, despite its use in the US [5–7], it remains unapproved in South Korean, resulting in limited availability of medical abortion [8]. - In the US, the Food and Drug Administration (FDA) has taken a proactive, two-track approach to increasing access to medial abortion by approving a generic version of mifepristone, - Even with these precautions, concerns persist safety of mifepristone, particularly risks of severe bleeding, ectopic pregnancy, and systemic infections [10,11]. | | **[Location: Introduction section]**   - In contrast, South Korea has yet to approve mifepristone despite the 2019 Constitutional Court ruling decriminalizing abortion up to 14 weeks of gestation [12,13]. - *(deleted)* - In the US, despite the FDA’s regulatory oversight and the adoption of Risk Evaluation and Mitigation Strategy, reports of adverse events such as severe bleeding, ectopic pregnancy, and systemic infections persist [7–11]. |
| **Reviewer’s Comment** | Moreover, the legal and regulatory references appear somewhat imbalanced: while the US context is discussed in detail, the Korean legal framework receives more limited attention. A more thorough and globally contextualized background would be beneficial. In particular, integrating European examples would offer a more comprehensive overview of international approaches to medical abortion—especially since the conclusions highlight global policy implications. To that end, I recommend including references such as:  https://pubmed.ncbi.nlm.nih.gov/34897104/  https://pubmed.ncbi.nlm.nih.gov/12831608/  https://pubmed.ncbi.nlm.nih.gov/12137129/  These sources can enrich the comparative dimension of the analysis and support a more robust global framing. | |
| **Before (Revision 1)** | | **After (Further revised version)**  We have further elaborated the introduction section to provide a more thorough and globally contextualized background, including European examples, to offer a more comprehensive overview of international approaches to medical abortion as follows. |
| **[Location: Introduction section]**  Mifepristone, a common oral abortifacient, is legally available in numerous countries, and the World Health Organization (WHO) has endorsed the combined use of mifepristone and misoprostol as safe, effective, and highly acceptable regimen for early abortion—defined as abortion during the first trimester [3]. In 2019, the WHO moved these medications from the “complementary” to the “core” list of its Essential Medicines List, given their critical role in global reproductive health [4].  Following these recommendations, the global prevalence of medical abortion has increased, particularly in high-income countries [5]. By 2017, medical abortion comprised more than one-half of all abortion provisions in at least 24 high-income nations, with high proportions in Sweden, England, and the United States (US). In the US in 2023, approximately 63% of all abortions were medication-based abortions [5]. Nevertheless, regulatory approval and access to mifepristone vary among countries; for instance, despite its use in the US [5–7], it remains unapproved in South Korean, resulting in limited availability of medical abortion [8].  In the US, the Food and Drug Administration (FDA) has taken a proactive, two-track approach to increasing access to medial abortion by approving a generic version of mifepristone, while simultaneously addressing safety concerns through a Risk Evaluation and Mitigation Strategy (REMS) [9]. The REMS program includes healthcare provider certification, comprehensive patient education, and post-marketing surveillance monitoring to address potential risks associated with mifepristone use. Even with these precautions, concerns persist safety of mifepristone, particularly risks of severe bleeding, ectopic pregnancy, and systemic infections [10,11].  Recently, public advocacy has increased to ensure mifepristone’s market availability in the US [12]. Meanwhile, in South Korea, mifepristone remains unapproved and inaccessible via legal channels [13] for reasons that include cultural stigma associated with abortion, limited public discourse surrounding reproductive rights, and the prevailing influence of conservative societal values [14]. However, these restrictions have raised debates about women’s health, underscoring the need for evidence-based policies prioritizing reproductive autonomy [15]. | | **[Location: Introduction section]**  Approved by the United States (US) Food and Drug Administration (FDA) in 2000 after several decades of use in European countries such as France, the United Kingdom. Italy and Sweden, mifepristone is now available in numerous countries, following the World Health Organization’s (WHO) endorsement of its use in combination with misoprostol as an early abortion regimen [3−6]. Prevalence of medical abortion has increased worldwide, comprising more than half of all abortions in high-income countries. In the US, despite the FDA’s regulatory oversight and the adoption of Risk Evaluation and Mitigation Strategy, reports of adverse events such as severe bleeding, ectopic pregnancy, and systemic infections persist [7–11]. In contrast, South Korea has yet to approve mifepristone despite the 2019 Constitutional Court ruling decriminalizing abortion up to 14 weeks of gestation [12,13]. Cultural stigma, conservative values, and limited public discourse on reproductive ethics continue to restrict access, underscoring the need for cautious, evidence-based policymaking than balances women’s health with broader moral and societal consideration [13]. |
| **Before (Revision 1)** | | **After (Further revised version)**  We revised the introduction section using the references recommended by the reviewer, as follows. |
| **[Location: References section]**  (None) | | **[Location: References section]**  3. Jones RK, Henshaw SK. Mifepristone for early medical abortion: experiences in France, Great Britain and Sweden. Perspect Sex Reprod Health. 2002;34(3):154–61.  4. Arisi E. Changing attitudes towards abortion in Europe. Eur J Contracept Reprod Health Care. 2003;8(2):109-21. |
| **Reviewer’s Comment** | The initial number of articles retrieved per country (prior to filtering or selection) is not explicitly stated—this detail would improve transparency. Additionally, I would suggest adding a brief note regarding the reproducibility of the research process (e.g., availability of code, corpora, or full protocol), which is particularly important in computational text analysis. | |
| **Before (Revision 1)** | | **After (Further revised version)** |
| **[Location: Method section]** The search words included “Mifepristone,” “Misoprostol,” “Mifegyne,” and “RU-486” in Korean news platforms and in English for the US news platforms.  **[Location: Results section]** Following the text analysis pipeline described in Fig 1, we analyzed 7,938 Korean and 650 US news articles on medical abortion with mifepristone published between 2006 and 2022. | | **[Location: Method section]** The search included news articles that contained the keywords “Mifepristone,” “Misoprostol,” “Mifegyne,” and “RU-486” in either the title or the main context. On the Korean news platforms these keywords were searched in Korean, while English terms were used for the US news platforms.  **[Location: Results section]** Following the text analysis pipeline described in Fig 1, duplicate articles were automatically removed using a pre-specified script, after which all remaining articles were manually reviewed; no further exclusions were required. A total of 7,938 Korean articles and 650 US articles on medical abortion with mifepristone, published between 2006 and 2022, were included in the final analysis.  **[It has been added at the end of the manuscript]** **Data Availability Statement**  The datasets analyzed in this study were derived from publicly available online news articles retrieved from Google News and Naver News between January 2006 and December 2022. Both the processed datasets and the analysis code used in this study are available from the corresponding author upon reasonable request. However, the exact number of accessible articles or the composition of the corpora may vary in the future depending on changes in news outlet availability or platform crawling policies. |
| **Reviewer’s Comment** | To further support interpretation, I suggest including a short summary section that explicitly highlights the key differences observed between the two countries in terms of themes and sentiments. | |
| **Before (Revision 1)** | | **After (Further revised version)** |
| **[Location: Discussion section]** This study …, we identified prominent topics, underlying sentiments, and word associations that illustrate public attitudes and policy discourse regarding medical abortion within regulatory and healthcare contexts. | | **[Location: Discussion section]** This study …, we identified prominent topics, underlying sentiments, and word associations that illustrate public attitudes and policy discourse regarding medical abortion within regulatory and healthcare contexts.  The findings revealed media coverage in South Korea primarily emphasized the regulatory approval process, safety concerns, and social controversies. In contrast, the US media discourse was characterized by legal debates, clinical applications, and accessibility, with more polarized sentiment patterns. |
| **Reviewer’s Comment** | In the Discussion, two aspects warrant careful attention:  Avoid implying causal relationships that are not empirically grounded. For example, the assumption that illegal online trade results directly from regulatory restrictions should be carefully qualified as a possible causal link or interpretative hypothesis, unless further evidence is provided.  The influence of media narratives on public opinion is mentioned but not sufficiently explored. A brief reflection on how and why media framing can shape public understanding—supported by relevant literature—would add theoretical depth and policy relevance to the discussion. | |
| **Before (Revision 1)**  **[Location: Discussion section]** | | **After (Further revised version)**  **[Location: Discussion section]** |
| - Such reporting may reflect unintended consequences of stringent regulatory controls, inadvertently fostering unsafe practices and misuse and thereby raising public health and safety concerns. - This omission suggests a strong media focus on practical considerations related to the introduction of medical abortion rather than on legal debates surrounding the abolition of abortion laws. - This practice may indicate the evolving nature of public discourse on abortion in South Korea; it merits a further investigation into how media framing shapes the public understanding of complex health concerns. | | - Such reporting may suggest possible unintended associations between stringent regulatory controls and unsafe practices and misuse, raising public health and safety concerns [34]. - This omission may indicate a strong media focus on practical considerations related to the introduction of medical abortion rather than on legal debates surrounding the abolition of abortion laws. - The media narratives are becoming increasingly important in shaping public belief and opinions, ultimately contributing to social change [14,35]. This practice may indicate the evolving nature of public discourse on abortion in South Korea, warranting further investigation into how media framing shapes the public understanding of complex health concerns. |
| **Reviewer’s Comment** | The Conclusion successfully summarizes the findings but remains rather general. It would benefit from being more precise and action-oriented, particularly by offering concrete recommendations. For example, the authors could suggest how media coverage might adopt a more balanced and informative narrative regarding medical abortion, or how policymakers might engage with media discourses more strategically. | |
| **Before (Revision 1)**  **[Location: Conclusion section]** | | **After (Further revised version)**  **[Location: Conclusion section]** |
| This study identifies ~ in each country. The topic modelling results showed that the Korean news focuses on regulatory challenges, safety concerns, and social perceptions, whereas the US coverage underscores clinical aspects and access-related debates.  Our findings reveal ~ surrounding medical abortion. | | This study identifies ~ in each country. The Korean news coverage focused on regulatory challenges, safety concerns, and social perceptions, whereas the US coverage underscored clinical aspects and access-related debates.  Our findings reveal ~ surrounding medical abortion through media discourses. Furthermore, it is essential for the media to adopt more balanced and informative narratives when addressing culturally and ethically sensitive topics, including medical abortion. |
